# The development of a Patient Decision Aid and Patient Concerns Inventory for people diagnosed with recurrent head and neck cancer: a mixed-methods study protocol

**DOI:** 10.1136/bmjopen-2026-116238

**Published:** 2026-07-02

**Authors:** Sofia Georgopoulou, Grainne Brady, Mark Fuller, Michael Thick, Susanne Cruickshank, Vinidh Paleri, Justin Roe

**Affiliations:** 1 Applied Health Research Group, The Royal Marsden NHS Foundation Trust, London, UK; 2 The International Centre for Recurrent Head and Neck Cancer (IReC), London, England, UK; 3 Department of Speech, Voice and Swallowing, The Royal Marsden NHS Foundation Trust, London, UK; 4 Head and Neck Unit, The Royal Marsden NHS Foundation Trust, London, UK; 5 The Institute of Cancer Research, London, England, UK; 6 Department of Surgery and Cancer, Imperial College London, London, UK

**Keywords:** Recurrent head and neck cancer (rHNC), treatment decision-making, unmet needs, Patient Decision Aid (PDA), Patient Concerns Inventory (PCI)

## Abstract

**Introduction:**

Historically, patients diagnosed with recurrent head and neck cancer (rHNC) had a poor prognosis with the majority receiving best supportive care. Significant treatment advances over the past decade have changed the landscape for curative and non-curative management of rHNC with more treatment options for patients. This requires providing large amounts of information about treatment options and patients may experience different short-, medium- and long-term toxicities. To address complex discussions on treatment decisions and to identify potential unmet needs and provide timely support, the study aim is to co-produce a bespoke Patient Decision Aid (PDA) and a Patient Concerns Inventory (PCI) for the rHNC population.

**Methods and analysis:**

This study uses a five-stage, multiple methods design. Stage 1 will include the creation of a steering group and Stage 2, a systematic review and evidence synthesis. Stages 3 and 4 are conducted as two parallel workstreams. The development of the PDA (Workstream 1) includes patient interviews and focus groups and that of the PCI (Workstream 2) patient focus groups and a Delphi survey for clinicians. Both workstreams conclude in Stage 5 with the steering group review and final comments. Patient and public involvement and engagement representatives will work in partnership with the study team and the steering group throughout the study. Analysis will include descriptive statistics and thematic analysis. This work will culminate in the development of a prototype Patient Decision Aid for patients with rHNC (PDA HN-R) and a Patient Concerns Inventory for patients with rHNC (PCI HN-R).

**Ethics and dissemination:**

All participants will receive detailed study information and give written informed consent before data collection. Ethical approval was obtained from the Health Research Authority, Research Ethics Committee and National Health Service (NHS) Research and Development Departments. All data collection will follow all legislative rules regarding data protection, also following the Declaration of Helsinki. Study results will be disseminated in peer-reviewed journals, presented at international conferences and charities.

**STRENGTHS AND LIMITATIONS OF THIS STUDY**

- Co-production of a bespoke Patient Decision Aid and Patient Concerns Inventory.
- Multiple methods design involving patients with recurrent head and neck cancer (rHNC) and healthcare professionals (HCPs).
- Maximised inclusivity and diversity in recruitment using various strategies.
- Relatively tight timeline for completion of the study.
- Participating centres are specialised so data might not be generalisable across other populations.

## Introduction

 Head and neck cancer (HNC) is treated using one or a combination of modalities including radiation, surgery and immunotherapy, based on the primary site and stage. These treatments can cause profound, long-term functional changes affecting function including eating, drinking and swallowing, speech, voice, mouth opening and subsequent overall quality of life (QoL).^1^ Unfortunately, nearly 40% of patients experience recurrence.^2^

### Recurrent head and neck cancer

Until recently, patients with recurrent HNC (rHNC) had a very poor prognosis with the majority of patients receiving only best supportive care.^3^ However, over the past decade there have been rapid advances in the treatment of rHNC. A landmark systematic review and meta-analysis investigated survival rates for those undergoing curative treatments including surgery and re-irradiation.^4^ This study revealed a 5-year survival rate of just 18% for those treated curatively prior to the year 2000, which increased to 51% from the year 2000 onwards. This has led to a paradigm shift in the management of rHNC with more patients being considered for curative treatments including surgery or re-irradiation.^5^ Additionally, minimally invasive surgical approaches have shown efficacy in the management of select rHNC, thus increasing the repertoire of procedures available for cure.^6^ The increase in options for surgical intervention and complexity of perioperative management has led to consensus recommendations being set out for this patient group.^7^

In parallel to this, there have also been significant changes in the treatment of patients who have inoperable disease, where re-irradiation is not feasible (non-curable disease). Historically these patients, if medically fit enough, would have been offered chemotherapy with an average prognosis of 8 months^8^ or best supportive care.^5^ Two landmark trials KEYNOTE 040^9^ and KEYNOTE 048^10^ have resulted in the adoption of immunotherapy as standard treatment for patients with inoperable disease, with a clinically meaningful survival benefit over standard chemotherapy regimens. Long-term follow-up has also shown 30% survival at 4 years in patients who are selected based on the biological markers of their tumour.^11^ These results have collectively changed the treatment landscape for curative and non-curative management of rHNC. Indeed, new studies are ongoing into new novel therapeutics for patients with rHNC with some promising early results.^12^ While this is encouraging for people diagnosed with rHNC, it brings with it a new set of challenges: providing large amounts of information about treatment options and short-, medium- and long-term toxicities on a background of already compromised function from primary disease treatment.

### Proposed instruments for supporting decision-making and identification of patient concerns in rHNC

Professionals lack the tools to support patients with complex decisions before treatment and to address ongoing QoL concerns and needs following treatment. To address these complex discussions of treatment decisions and to identify potential unmet needs and provide timely support, a Patient Decision Aid (PDA) and a Patient Concerns Inventory (PCI) bespoke for the recurrent HNC population are essential.

PDAs are condition-specific, decision-making tools that facilitate shared decision-making by providing plain-language information about the potential risks (short- and long-term side effects), benefits and uncertainties related to treatment, and information on how QoL and daily life could be affected.^13^ PDAs can be in the format of a webpage, pamphlet or video/audio. Currently, there are no PDAs for patients with rHNC. This highlights the need for this research to design and develop a PDA tailored to the needs of this particular patient group.

PCIs are condition-specific prompt lists of potential issues patients might wish to raise in discussion with the clinical team which otherwise, although being very important to the patient, could be missed or overlooked. PCIs can be paper-based or electronic and are self-administered by a patient. While generic holistic needs assessment tools are widely used in cancer services, both the literature and our consultations with our patient and public involvement and engagement (PPI/E) groups, concur that these generic checklists do not meet the unique needs of people diagnosed with rHNC.

### Existing tools for the identification of concerns in HNC

There are two current PCI versions available for patients with HNC, currently for use after diagnosis and for use after treatment.^14^ However, the initial validation and testing of these tools was completed with patients with primary disease, excluding those with recurrence. Recent literature has identified that patients with disease recurrence are a distinct entity in comparison to those with primary disease as they are experienced patients with HNC with unique physical, psychological and emotional needs.^15^ Patients with rHNC often present with persistent, late toxicities from previous treatment, including difficulties with eating and drinking and communication.^16^ Patients who have been treated for HNC also have a high level of psychological burden with increased rates of anxiety and depression.^17^ Second, initial development of the PCI-HN was completed in 2009, based on the needs of patients treated with open surgery and/or chemoradiation. Since 2009, the landscape of treatment of recurrent HNC has changed dramatically with the advent of minimally invasive surgery^6^ and immunotherapy treatments.^9 10^ It continues to evolve with clinical trials of novel agents.^12^ Consequently, a PCI, specific to this population, which caters to their specific needs, offers an opportunity to address both physical and mental health for the HNC recurrence population which are different from those patients with primary HNC disease.

### The expected benefits of the PCI HN-R and PDA-HN-R

The development of a Patient Concerns Inventory for patients with rHNC (PCI HN-R) could provide a tool for use in clinical practice so that the specific concerns of patients can be identified by patients and addressed by healthcare professionals. In previous research, the PCI-HN has been shown to be an effective screening tool to identify those patients at high risk of poor health-related QoL.^18^ There could be a similar benefit with a tool developed for patients with recurrent disease.

When the newly developed Patient Decision Aid for patients with rHNC (PDA HN-R) is used at diagnosis, we hope that it will help with decision-making, understanding outcomes and their expectations regarding their treatment and care going forward, thus enhancing shared decision-making. The PDA HN-R will provide patients with information about the available treatments for rHNC in a way that is accessible to them. With evolving treatments and evidence base, PDA HN-R can be adapted to subsequent generations of patients.

The needs of patients with rHNC are also likely to change over time, for example before and after treatment. Therefore, the PCI HN-R can be repeated at every consultation to ensure the patients’ needs are being identified and met.

### Aims and objectives of the study

#### Aim

To co-design two self-administered instruments for people diagnosed with rHNC: one that will aid decision-making before treatment and another to elicit concerns and priorities after diagnosis and during follow-up visits after treatment.

#### Objectives

1. To develop a prototype PDA HN-R.
2. To develop a prototype PCI HN-R.

## Methods and analyses

### Study design

**Figure 1:**
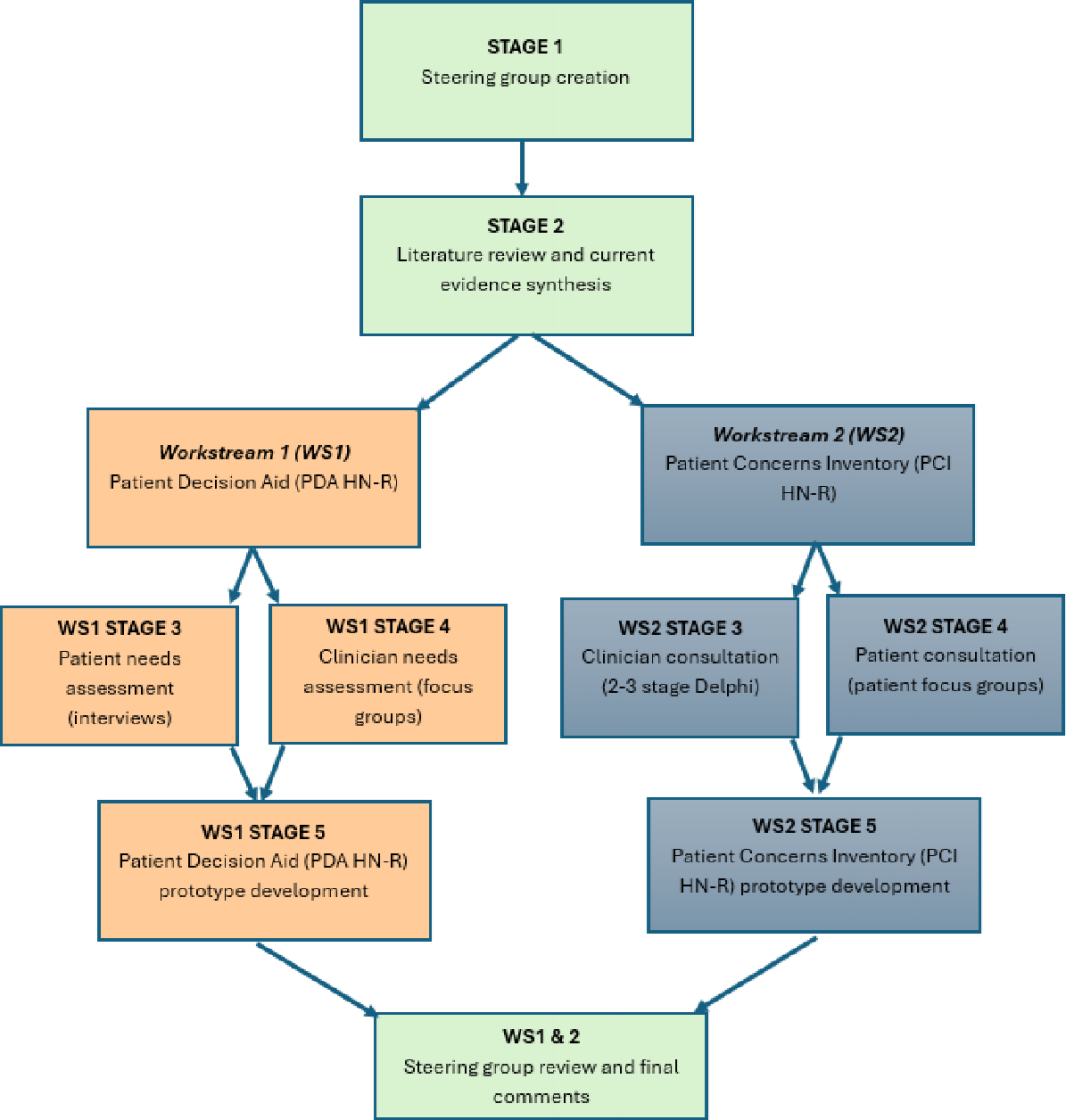
Overview of the CONSIDER study design. CONSIDER, The development of a Patient CONcernS Inventory and DEcision aid for patients with Recurrent HNC; PCI HN-R, Patient Concerns Inventory for patients with rHNC; PDA HN-R, Patient Decision Aid for patients with rHNC; rHNC, recurrent head and neck cancer; WS1, Workstream 1; WS2, Workstream 2.

This is a 24-month, multiple methods, multi-site study with two interlinked workstreams (figure 1) involving five key stages. This study will adopt a co-design approach where patients and healthcare professionals work together as equal partners in prototype development. Workstream 1 will develop a PDA in accordance with the systematic process proposed by the International Patient Decision Aids Standards (IPDAS) and its quality criteria.^19^ Workstream 2 will be in accordance with the systematic process used in existing PCI development.^20^

### Population and setting

The study will include healthcare professionals (HCPs) and patients from the National Health Service (NHS) in the UK as part of the study population. HCPs and patient participants will be recruited across the International Centre for Recurrent Head and Neck Cancer (IReC) collaborative network. The IReC network includes nine high-volume sites across the UK that regularly manage rHNC, representing a wide geographical spread and experience.

Patients will be invited to participate in the study by their care team when they attend routine outpatient follow-up appointments. HCPs will be invited to participate via email. To maximise inclusivity and diversity in recruitment, the study will (a) recruit participants across England via the IReC Network and (b) cover costs for the research team to travel to the participants, for interpreters as needed, and for caring responsibilities for patients with dependents. We will ensure that options are kept open for both virtual and face-to-face forums for focus groups and interviews.

### Study procedures

The first two stages include the creation of a steering group (Stage 1) and a systematic review with evidence synthesis (Stage 2). Stages 3 and 4 are conducted as two parallel workstreams (1 & 2). Workstream 1 concentrates on the development of the PDA HN-R which comprises patient interviews and focus groups to assess HCPs’ needs. Workstream 2 focuses on the development of the PCI HN-R and involves patient focus groups and a Delphi survey for clinicians. Both workstreams conclude in Stage 5 with the prototype development and the steering group review to finalise the instruments. PPI/E representatives will work in partnership with the study team and the steering group consulting throughout the study.

### Workstreams 1 and 2—stage 1: steering group assembly (February–March 2026)

A multidisciplinary team (MDT) steering group of allied health professionals (AHPs), HNC surgeons, oncologists, HNC specialist nurses and two expert patients will be formed to lead the study, identify potential study participants and assist in designing and reviewing the PDA and PCI prototypes. The patients and HCPs will be approached via the IReC network.

### Workstreams 1 and 2—stage 2: systematic review and current evidence synthesis (February–September 2026)

#### Aim

Stage 2 will identify baseline metrics for informing PDA and PCI prototype development by undertaking a synthesis of systematic reviews and national datasets and guidelines. A baseline for survival and functional outcomes will be established to inform the information section of the PDA, using:

1. A recently established consensus for the management of recurrent head neck cancer across the domains of salvage surgery including prehabilitation, rehabilitation and nutrition with up-to-date data on outcomes from surgical salvage.
2. Metrics for oncological outcomes after non-surgical treatment from recent trials, the international guidelines for cancer care and the expertise within the IReC network. Where gaps in our knowledge exist, the systematic review will be performed to identify the baseline.

Additionally, the consensus is being regularly revised by IReC as new data are being acquired which will be included in the PDA to reflect recent advances following consultation with the project steering group and patient representatives.

### Workstream 1—stages 3 and 4: needs assessments with patients/clinicians (October 2026–July 2027)

#### Aim and design

The aim of these stages for the development of the PDA is to explore the decisional needs from a patient’s and a clinician’s perspective regarding the treatment decision-making between curative and non-curative treatment for rHNC. A qualitative study using in-depth patient interviews and focus groups with HCPs will be undertaken for this purpose. The topic guide for both the patient interviews and HCP focus groups will be developed with the project patient collaborators.

#### Setting

HCPs and patient participants will be recruited across the IReC collaborative network. The IReC network includes sites across the UK representing a wide geographical spread and the experience of many different centres. A minimum of two centres will be approached, and collaborative agreements will be put in place so that these locations can function as Participant Identification Centres (PICs) for this study. We have identified two PIC sites that are willing to participate and are in different geographical areas in order to capture potential demographic and clinical differences. Regarding participants, there will be two separate samples—one for the PCI and another for the PDA as they have different eligibility criteria. The eligibility criteria for the PDA and PCI are different because the PDA is for patients where there is a decision between curative and non-curative treatment whereas the PCI can be used for all patients regardless of treatment type/pathway. Recruitment will extend across three large, specialised centres. Thus, it is not anticipated that the separate samples of participants will have a negative impact on achieving the target numbers, especially as the target number is reasonably low.

#### Recruitment and consent

Patients will be invited to participate in the study by their care team when they attend routine outpatient follow-up appointments. They will be provided with a written participant information leaflet. If the patient participant expresses interest in being involved, their usual care team will advise them that if they agree, the research team will contact them via their preferred communication method (letter, email, telephone call). A member of the research team will then contact the patient, confirm that they wish to be involved and schedule an interview (either face-to-face or virtual) depending on the participant’s preference. The information contained in the participant information sheet (PIS) will be revised. Informed consent to take part in the study will be obtained in person or online by a member of the research team and an interview will be scheduled at a time that is convenient to the participant. Participants will be made aware that they can withdraw from the study at any time and that their study data will not be included. Participants will be reassured that this will not impact on their clinical care. Demographic information will be collected at the time of the interview. Interviews will be conducted using a topic guide developed with the PPI/E collaborators. Interviews will be audio- recorded with permission. If a potential participant is identified at one of the study’s PICs, their usual care team will provide them with a study leaflet and advise them to contact the research team directly should they wish to be involved.

HCPs will be invited to participate via email. A PIS will be sent with the email invitation. HCPs will be asked to express their interest in participating via return email. Any interested HCPs will then be invited to an online focus group. Written informed consent will be obtained prior to the focus group. Demographic information will be collected at this time, including profession and time since diagnosis of rHNC. HCP focus groups will be audio-recorded with permission.

### Workstream 2—STAGE 3 and 4: 2–3 stage Delphi and focus groups with patients (October 2026–July 2027)

#### Aim and design

A multidisciplinary HCP online Delphi survey will be completed. The proposed item list generated from the systematic review, in addition to any items included in the existing PCI-HN tool for patients with primary HNC, will be modified based on the responses from the MDT members. Online patient focus groups will also be completed, where participants will be asked to comment on the content, form and use of the PCI HN-R.

#### Setting

HCPs and patient participants will be recruited across the IReC collaborative network.

#### Recruitment and consent

Recruitment of patient participants will follow the same process as above. To reduce the burden on participants involved in Workstream 1, additionally, new participants will be invited. Focus groups will be conducted either in person or virtually, using a topic guide developed with the PPI/E collaborators. Focus groups will be audio-recorded with permission. HCPs will be invited to take part in the Delphi study via an email invitation containing a PIS. Once the HCP has confirmed informed consent electronically, they will send the link to the online survey with a specified deadline for completion. At each stage of the Delphi, this process will be completed.

### Workstreams 1 and 2—stage 5: prototype development (August 2027–January 2028)

Based on the assessment of needs of key stakeholders and available evidence from existing literature, a prototype PDA HN-R will be drafted according to the criteria for developing decision aids and assessed using the International Patient Decision Aid Standards instrument (IPDASi) V.4.0.^21^ As per the IPDAS guidelines,^22^ the content of the PDA HN-R will include four main topics:

1. Brief descriptions about the disease and treatment options.
2. Possible treatment outcomes.
3. Risks and benefits of treatment options.
4. A value clarification exercise.

The IPDAS checklist will ensure the content of the PDA and the development process meet its quality criteria. The combined findings of the online MDT survey and patient focus groups will guide the development of a prototype PCI HN-R. Once the prototypes have been developed, they will be reviewed by the steering committee, including professionals and patients, with confirmation of face validity in preparation for further user testing.

### Eligibility criteria: workstream 1 (PDA HN-R)

#### Patients

To be eligible to participate in the development of the PDA HN-R in this study, a patient must meet all the following inclusion criteria:

1. Age 18 or above.
2. Competency to provide consent.
3. Adequate linguistic and cognitive skills to take part in an interview.
4. Biopsy-proven locoregionally rHNC.
5. Head and neck multidisciplinary team meeting discussion documenting >1 potential management option.

#### Healthcare professionals

To be eligible to participate in the development of the PDA HN-R in this study, an HCP (surgeon, oncologist, AHP, nurse) must meet only the following inclusion criterion:

- A minimum of 5 years’ clinical experience in working with patients with rHNC.

### Eligibility criteria: workstream 2 (PCI HN-R)

#### Patients

To be eligible to participate in the development of the PDA HN-R in this study, a patient must meet all the following inclusion criteria:

1. Biopsy-proven locoregionally recurrent HNC.
2. Undergoing systemic treatment (immunotherapy/chemotherapy/clinical trial of investigational agents) or have undergone surgery and/or re-irradiation for HNC recurrence.

#### Healthcare professionals

To be eligible to participate in the development of the PDA HN-R in this study, an HCP (surgeon, oncologist, AHP, nurse) must meet only the following inclusion criterion:

- A minimum of 5 years’ clinical experience in working with patients with rHNC as with the PDA.

### Data analysis

#### Sample size calculation

For stages 3 and 4, a sample size of 10–15 patients and 10–15 HCPs for each stage and each workstream is typically considered sufficient to achieve data saturation in qualitative research.^23^ HCPs will include a range of professionals, at least two from each professional group, that is, surgeons, oncologists, AHPs and nurses; however, we will follow the principle of data saturation, i.e., data collection will be continued until no new themes or insights emerge from the interviews or focus groups.

#### Qualitative analysis

The qualitative data collected from focus groups with HCPs and patients’ interviews will be analysed using a structured approach. The audio-recorded data will be transcribed and organised before undergoing a rigorous coding process to identify recurring themes and patterns. The qualitative analysis software (QSR NVivo 12)^8^ will be used for coding based on emergent themes, concepts and categories using the Framework Approach.^22^

#### Quantitative analysis

Delphi survey results will be analysed quantitatively, reporting percentage consensus agreement using GraphPad Prism Software. The combined findings of the Delphi and patient focus groups will be used to adapt the existing PCI-HN tool to the needs of patients with rHNC. The use of this Delphi method with involvement of both HCPs and patients will ensure the content validity of the PCI HN-R.

### Patient and public involvement

#### Pre-proposal PPI

This study has been developed by patients, clinicians and researchers working together within the IReC collaborative. The research group was awarded a PPI/E grant to inform the initial planning of this project via the NIHR Biomedical Research Centre at the Royal Marsden and the Institute of Cancer Research. The specific funding twice daily call for this PPI/E funding call was focused on reaching out and collaborating with diverse communities and new patient and public contributors. As a result of this grant, we were able to extend the membership of the IReC PPI/E group, which highlighted the development of tools to aid decision-making and reporting of concerns. They have already reviewed existing tools and emphasised how new tools are required to meet the unique needs of patients with rHNC. For this proposal, the lay summary and full proposal have been reviewed by our patient collaborators (MF and PD). MF and another patient collaborator MT have expressed their desire to be part of the project steering committee. A further two members of the public with no personal experience of HNC also reviewed the plain English summary.

The PPI/E collaborators who will be recruited to the project steering group to work alongside the professionals will be consulted on specific areas throughout the research. As with all research undertaken at RM, any PPI/E activities will be undertaken under the guidance set by the NIHR. All PPI/E members underwent PPI/E induction training which aims to provide an overview of patient and public involvement at The Royal Marsden. The PPI/E collaborators for this project will also have a bespoke training session with the research team on PCI and PDA development methodology. PPI/E collaborators will help with the preparation for participant materials to ensure that they are clear and understandable prior to submission for HRA approvals.

At the end of each stage of the research, the findings will be relayed to them for discussion to ensure they are in line with their own experience. PPI/E team will also be key in dissemination of findings including the design of clear infographics and via social media to ensure that patients and members of the public are aware of the research and will co-author publication outputs. The PPI/E budget has carefully been planned so that the PPI/E contributors are compensated for their time and expertise in line with the guidelines of the NIHR.

#### PPI/E During the project

The project adopts a co-design methodology guided by principles of sharing power; including all perspectives and skills, respecting and valuing the knowledge of all those working together; reciprocity and building and maintaining relationships. The PI will lead and coordinate all PPI activity with the guidance of co-applicants with lived experience and will follow the UK Standards for Public Involvement. This work has been costed for within the budget and will cover both patient and public activity.

### Further work

The research team are already planning for further evaluation of our PDA HN-R and PCI HN-R prototypes to complete their user and pilot testing.

## Ethics and dissemination

### Ethics

The study will be conducted in compliance with the principles of the Declaration of Helsinki (2013),^19^ the Principles of Good Clinical Practice and in accordance with the Research Governance Framework for Health and Social Care (England).^24^ The study was approved by the London - Brent Research Ethics Committee and Health and Care Research Wales (HCRW) (REC reference: 26/PR/0114, registered on 25 February 2026).

### Ethical considerations

There are ethical concerns associated with approaching people living with rHNC for interview or focus groups, both in protecting their confidentiality and handling the sensitive subject matter that might be discussed. Consultation with patient representatives has guided our approach. Due to the nature of the study and its potential sensitivity, the researcher will be aware that the issues discussed may result in the participants feeling emotionally drained or upset. The protection of the participants’ overall well-being, as well as the confidentiality of the information they share, will be emphasised to them in writing and verbally. Should a participant become distressed during an interview, we will offer to stop, either to provide a break or completely, depending on their preference. We will work closely with the clinical team at the site in advance to identify an appropriate route for escalation and support resources if required. If they decline to be part of the study or withdraw, they will be reassured that this will not affect their care delivery in any way and that the researchers fully understand if this should happen. Participants can stop being part of the study at any time, without giving a reason, but any information about them that we already have will be kept. Transmission of data will only be undertaken by authorised staff.

### Dissemination plan

The data will be analysed and tabulated, and a final study report will be prepared and submitted to NIHR in line with their requirements and stipulated timelines. It will be prepared for publication in an open access journal within 12 months of completion of this study. The participants of the interviews and discussion groups will be asked to consent to the use of anonymised quotes being used in the report/publication. The publication will acknowledge the Royal Marsden as the study sponsor and the NIHR as the funder of this study, and publication of these results will follow NIHR guidance and the standard RMH policy for publication of research. All study participants will be provided with a copy of the study findings. The interview transcripts will not be publicly available, but the analysis tables will be included in publications either as primary or supplementary data and therefore will be publicly available.

### Status and timeline of the study

The study is expected to start in February 2026. Stages 1 and 2 are expected to last 8 months, stages 3 and 4 a further 10 months and stage 5 6 months. It is anticipated that the study will be completed by January 2028 with dissemination intended to be completed by January 2028 (online supplemental file 1).

## Supplementary material

10.1136/bmjopen-2026-116238 online supplemental file 1

## Data Availability

No data are available.
