# Supplementary material for: The development of a Patient Decision Aid and Patient Concerns Inventory for people diagnosed with recurrent head and neck cancer: a mixed-methods study protocol
Source: BMJ Open. 2026 Jul 2;16(7):e116238. doi: 10.1136/bmjopen-2026-116238 (PMC13330957; doi:10.1136/bmjopen-2026-116238)
Supplement: online supplemental file 1 [file bmjopen-16-7-s001.docx]

**Supplementary Materials – GANTT Chart CONSIDER Study**
