# Supplementary material for: The development of a Patient Decision Aid and Patient Concerns Inventory for people diagnosed with recurrent head and neck cancer: a mixed-methods study protocol
Source: BMJ Open. 2026 Jul 2;16(7):e116238. doi: 10.1136/bmjopen-2026-116238 (PMC13330957; doi:10.1136/bmjopen-2026-116238)
Supplement: Supplementary file 2 [file bmjopen-2026-116238.reviewer_comments.pdf]

PEER REVIEW HISTORY

BMJ Open publishes all reviews undertaken for accepted manuscripts. Reviewers are asked to complete a checklist review form and are provided with free text boxes to elaborate on their assessment. These free text comments are reproduced below.

ARTICLE DETAILS

Title (Provisional)

The development of a Patient Decision Aid and Patient Concerns Inventory for people diagnosed with recurrent head and neck cancer: a mixed-methods study protocol

Authors

Georgopoulou, Sofia; Brady, Grainne; Fuller, Mark; Thick, Michael; Cruickshank, Susanne; Paleri, Vinidh; Roe, J W

VERSION 1 - REVIEW

**Reviewer**	**1**
**Name**	**Ozakinci, Gozde**
**Affiliation**	**University of Stirling, Division of Psychology**
**Date**	**29-Apr-2026**
**COI**	**None**

This mixed-methods study aims to co-develop tailored co-produced tools to support decision-making and address concerns for patients with recurrent head and neck cancer (rHNC).

The authors identify the existing PCI (although only one is cited (page 4, line 11-12). Could the authors elucidate further why the current measures could no t be updated or what specific items are missing or would need revising?

Could the need for PDA HN-R be articulated with evidence from patients/carers and HCPs?

Could the authors make it clearer in the text (it's clear in the figure) that the WS1's focus groups are for clinician needs assessment as there's a risk of being understood as not involving clinicians?

Literature review: Page 7 lines 3-22 could make it clearer why the review is focusing on those metrics. It's not coming out clearly enough why those metrics/datasets are being focused on.

Could you provide more information on the site selection through IReC?

Will you make it clearer to the participants that they don't have to take part in the second WS data collection? How will this affect the pool of participants for the WS2 data collection?

Maybe I'm missing something but why are the patient eligibility criteria for both WSs not the same?

Page 10, Lines 14-16: it's mentioned that the existing PCI-HN tool will be adapted for rHNC patients. Again, maybe I missed it earlier but could you please check and make it clear that the existing measure is being adapted?

The PPIE component is exemplary and clearly articulated.

Minor edits:

Introduction, line 13-14: could you clarify the first sentence: is it the case that they 'only' receive supportive care?

VERSION 1 - AUTHOR RESPONSE

Reviewer: 1

1. **The authors identify the existing PCI (although only one is cited (page 4, line 11-12).**

   Thank you for your very important observation. This reference has now been updated to an online reference where both tools can be access and downloaded on the *Merseyside Regional Head and Neck Cancer HaNC PCI Head and Neck Cancer website available at https://www.hancsupport.com/professionals/patient-concerns-inventory/pci-head-neck-cancer*

2. **Could the authors elucidate further why the current measures could not be updated or what specific items are missing or would need revising?**

   Thank you for your excellent question. This paragraph has now been updated to include additional references explaining how the current PCI was developed for and validated with patients with primary HNC (please see below). This paragraph highlights how the existing tools do not meet the unique needs of the recurrence population. There is also a statement regarding PPI/E review of the current tools which further highlighted the need for new tool development.

   "Recent literature has identified that patients with disease recurrence, are a distinct entity in comparison to those with primary disease as they are experienced HNC patient with unique physical, psychological and emotional needs[15] . Patients with rHNC often present with persistent, late toxicities from previous treatment, including difficulties with eating and drinking and communication[16]. Patients who have been treated for HNC also have a high level of psychological burden with increased rates of anxiety and depression[17]" …"Consequently, a PCI, specific for this population, which caters to their specific needs, offers an opportunity to

address both physical and mental health for the HNC recurrence population which are different from those patients with primary HNC disease".

3. **Could the need for PDA HN-R be articulated with evidence from patients/carers and HCPs?**

   That is a very helpful suggestion. This has been added as an additional section in relation to existing tools (please see comment #2).

4. **Could the authors make it clearer in the text (it's clear in the figure) that the WS1's focus groups are for clinician needs assessment as there's a risk of being understood as not involving clinicians?**

   That is an excellent point and will definitely help the reader understand that the focus groups are for HCPs. It has now been updated to read: *"Workstream 1 concentrates on the development of the PDA HN-R which comprises patient interviews and focus groups to assess healthcare professionals' needs."*

5. Literature review: Page 7 lines 3-22 could make it clearer why the review is focusing on those metrics. It's not coming out clearly enough why those metrics/datasets are being focused on.

   You are absolutely right and thank you for highlighting this short-coming. The literature review section has been updated to include details of the systematic review which is currently underway to inform the development of both of the tools and ow reads:

   Workstreams 1 & 2 – STAGE 2: Systematic review and current evidence synthesis (February – September 2026)

   Aim:

   Stage 2 will identify baseline metrics for informing PDA and PCI prototype development by undertaking a synthesis of systematic reviews and national datasets and guidelines. A baseline for survival and functional outcomes will be established to inform the information section of the PDA, using:

   1. *A recently established consensus for the management of recurrent head neck cancer across the domains of salvage surgery including prehabilitation, rehabilitation and nutrition with up-to-date data on outcomes from surgical salvage.*
   2. *Metrics for oncological outcomes after non-surgical treatment from recent trials, the international guidelines for cancer care and the expertise within the IReC network. Where gaps in our knowledge exist, systematic reviews will be performed to identify the baseline.*

6. **Could you provide more information on the site selection through IReC? Will you make it clearer to the participants that they don't have to take part in the second**

WS data collection? How will this affect the pool of participants for the WS2 data collection?

A very helpful suggestion. Thank you. We have identified two PIC sites that are willing to participate and come from different geographical areas in order to capture potential demographic and clinical differences. There will be two separate samples of participants as they have different eligibility criteria. However, due to recruitment extending across three large, specialised centres, it is not anticipated that the separate samples of participants will have a negative impact on achieving the target numbers especially as they are not particularly high. This has been clarified by adding the following section to Workstream A – Setting:

We have identified two PIC sites that are willing to participate and are located in different geographical areas in order to capture potential demographic and clinical differences. Regarding participants, there will be two separate samples – one for the PCI and on for the PDA as they have different eligibility criteria. The eligibility criteria for the PDA and PCI are different because the PDA is for patients where there is a decision between curative and non-curative treatment whereas the PCI can be used for all patients regardless of treatment type/pathway. Recruitment will extend across three large, specialised centres. Thus, it is not anticipated that the separate samples of participants will have a negative impact on achieving the target numbers especially as the target number is reasonably low.

7. **Maybe I'm missing something but why are the patient eligibility criteria for both WSs not the same?**

You are not missing anything at all. That is an excellent question. The eligibility criteria for the PDA and PCI are different because the PDA is for patients where there is a decision between curative and non-curative treatment whereas the PCI can be used for all patients regardless of treatment type/pathway. This has been added to the same section as comment #6 (please see above).

8. **Page 10, Lines 14-16: it's mentioned that the existing PCI-HN tool will be adapted for rHNC patients. Again, maybe I missed it earlier, but could you please check and make it clear that the existing measure is being adapted?**

That is a very good point. Just to clarify, only the PCI will be adapted to rHNC not the PDA as no such tool currently exists. This has been clarified by adding the following to Page 3 second paragraph: *"Currently, there are no PDAs for patients with rHNC. This highlights the need for this research to design and develop a PDA tailored to the needs of this particular patient group."*

9. **The PPIE component is exemplary and clearly articulated.**
   Minor edits: Introduction, line 13-14: could you clarify the first sentence: is it the case that they 'only' receive supportive care?

Thank you for your kind feedback. This sentence has now been updated to read: *“Until recently, patients with recurrent HNC (rHNC) had a very poor prognosis with the majority of patients receiving only best supportive care”*

The word count of the manuscript is 4,204 including the strengths and limitations section which exceeds the word limit. Unfortunately, this was inevitable as we needed to address the clarifications that were suggested by the reviewer.

We hope these revisions are satisfactory and address all the points you have raised.

Thank you again for taking the time to review this manuscript.

VERSION 2 - REVIEW

**Reviewer**	**1**
**Name**	**Ozakinci, Gozde**
**Affiliation**	**University of Stirling, Division of Psychology**
**Date**	**14-May-2026**
**COI**	

Thank you for addressing my queries and responding to my comments. Wish you all the best with your study.
